# The Key Role of c-Fos for Immune Regulation and Bacterial Dissemination in *Brucella* Infected Macrophage

**DOI:** 10.3389/fcimb.2018.00287

**Published:** 2018-08-21

**Authors:** Huynh T. Hop, Lauren T. Arayan, Tran X. N. Huy, Alisha W. B. Reyes, Son H. Vu, WonGi Min, Hu J. Lee, Man H. Rhee, Hong H. Chang, Suk Kim

**Affiliations:** ^1^Institute of Animal Medicine, College of Veterinary Medicine, Gyeongsang National University, Jinju, South Korea; ^2^College of Veterinary Medicine, Kyungpook National University, Daegu, South Korea; ^3^Institute of Agriculture and Life Science, Gyeongsang National University, Jinju, South Korea

**Keywords:** *Brucella abortus*, c-Fos, MAPKs, TLR-4, IL-10

## Abstract

The cellular oncogene c-Fos (c-Fos) is a component of activator protein 1 (AP1), a master transcriptional regulator of cells. The suppression of c-Fos signaling by siRNA treatment resulted in significant induction of TLR4, which subsequently activates p38 and ERK1/2 mitogen-activated protein kinases (MAPKs) and enhances F-actin polymerization, leading to an increase in *B. abortus* phagocytosis. During *B. abortus* infection, c-Fos signaling is induced, which activates the downstream innate-immunity signaling cascade for bacterial clearance. The inhibition of c-Fos signaling led to increased production of interleukin 10 (IL-10), which partially suppressed lysosome-mediated killing, resulting in increased survival of *B. abortus* inside macrophages. We present evidence of the regulatory role played by the c-Fos pathway in proliferation during *B. abortus* infection; however, this was independent of the anti-*Brucella* effect of this pathway. Another finding is the essential contribution of c-Fos/TRAIL to infected-cell necrosis, which is a key event in bacterial dissemination. These data provide the mechanism via which c-Fos participates in host defense mechanisms against *Brucella* infection and in bacterial dissemination by macrophages.

## Introduction

*Brucella* spp. are intracellular gram-negative bacteria that cause brucellosis in animals and in more than 500,000 human cases annually (Hop et al., 2017b). The virulence of *Brucella* spp. are thought to be due to the ability of these bacteria to prevent *Brucella* phagosome maturation by mechanisms that are not completely understood, resulting in successful proliferation within a number of phagocytes, such as macrophages, epithelial cells and placental trophoblasts, leading to chronic infection (Hop et al., 2017a; Reyes et al., 2017). Host resistance to *B. abortus* relies on the coordination of innate and adaptive immunity; thus, phagocytosis and subsequent processing of *Brucella* by macrophages are thought to be the major factors that drive this coordination and have important consequences for the control of initial infection and adaptive immunity activation (Kim et al., 2012). Therefore, the use of macrophages could be considered as an important tool to better characterize the immune response to *Brucella* infection. However, to date, very little is known about defense mechanisms activated in macrophages upon infection and about the successful virulence strategies used by *Brucella* to neutralize these responses for survival.

c-Fos belongs to Fos family and binds to c-Jun to form activator protein 1 (AP1), one of the most powerful transcriptional factors of the immune system (Chinenov and Kerppola, 2001; Shaulian and Karin, 2002). While AP-1 generally acts as an activator of pro-inflammatory genes, the function of c-Fos seems to be the opposite (Ray et al., 2006). In macrophages, c-Fos was demonstrated to suppress the expression of inducible nitric oxide synthase (*iNos*) and pro-inflammatory cytokines, including tumor necrosis factor (*Tnf*) and interleukins 6 and 12 (*Il6, Il12*), in conjunction with an increase in the expression of anti-inflammatory genes (*Il10, socs1*, and *socs3*). In addition, mice lacking c-Fos also exhibit a marked increase of pro-inflammatory cytokines in the context of *Salmonella* infection or endotoxin exposure, and the nuclear factor kappa B (NF-*k*B) pathway was shown to be involved in the regulatory role of c-Fos in macrophages and in a mouse model (Okada et al., 2003; Ray et al., 2006; Maruyama et al., 2007). Moreover, c-Fos was also proven to regulate apoptosis in different kinds of cells (Preston et al., 1996; Galea et al., 1999; Asim et al., 2010), suggesting a role for c-Fos in immune modulation.

Although various studies have suggested a role for c-Fos in inflammation, apoptosis and the immune system, the identity of the downstream molecular cascade activated/inhibited by the c-Fos pathway during bacterial infection remains unknown. Thus, in this study, using the intracellular bacterium *B. abortus* as a model, we attempted to elucidate the effect of c-Fos signaling on important immune effectors such as MAPKs, F-actin, TLR-4, cytokines, phagolysosome fusion and necrosis, which may provide insight into the fundamental role of c-Fos in the immune response against microbial infection.

## Materials and methods

### Reagents

Mouse *c-fos* siRNA, control siRNA-A, rat polyclonal anti-LAMP-2 and FITC-rabbit polyclonal anti-TLR4 antibodies were obtained from Santa Cruz Biotechnology (USA). Rat polyclonal anti-CtsA, anti-CtsL and rabbit polyclonal anti-CtsH antibodies were purchased from MyBioSource. Rabbit polyclonal anti-CtsC antibody was obtained from Antibodies-online, while rhodamine-phalloidin was purchased from Thermo Fisher Scientific (USA). Rabbit monoclonal anti-c-Fos, anti-phosphor-c-Fos (p-c-Fos), rabbit polyclonal anti-p-JNK, anti-p-ERK1/2, anti-p-p38, anti-JNK, anti-ERK1/2, and anti-p38 antibodies were purchased from Cell Signaling Technology (USA). Texas red-goat anti-rat IgG antibody and Lipofectamine RNAiMAX were purchased from Life Technologies (USA). Fluorescein isothiocyanate (FITC), FITC-conjugated goat anti-rabbit IgG antibody, lysophosphatidylcholine and tetramethyl rhodamine isothiocyanate-phalloidin (phalloidin-TRICT) were obtained from Sigma-Aldrich Corp (USA).

### Bacterial preparation and cell culture

The *B. abortus* 544 biovar 1 strain, provided by the Animal and Plant Quarantine Agency, Korea, was cultured in *Brucella* broth (BD Biosciences, USA) at 37°C for 3 days. Labeling of bacteria with FITC was performed as previously described (Nichols et al., 1993; de Boer et al., 1996; Reyes et al., 2017). Briefly, *B. abortus* (1 × 10^9^/ml) were washed with PBS, suspended with 0.5 ml of FITC in PBS (1 mg/ml) and incubated for 30 min under constant shaking at 37°C in the dark. FITC-labeled *Brucella* was washed three times with PBS prior to the internalization assay.

The macrophage RAW 264.7 cells were grown in RPMI 1640 containing 10% (v/v) heat-inactivated fetal bovine serum (FBS) with or without 100 U/ml penicillin and 100 μg/ml streptomycin at 37°C in 5% CO_2_.

### RNA interference

RAW 264.7 cells were grown to 50% confluence and transfected with *c-fos* siRNA using Lipofectamine RNAiMAX. The cells were incubated at 37°C in 5% CO_2_ for either 24 or 48 h prior to subsequent experiments. The same concentration of negative control siRNA was used in all experiments as a control. Knockdown efficiency was quantified by qRT-PCR.

### Proliferation assay

Cell proliferation was evaluated by the Rapid Cell Proliferation Kit in accordance with the manufacturer's instructions (EMD Millipore, USA).

### FACS assay for bacterial internalization and intracellular growth

Because *c-fos* siRNA treatment reduced cell proliferation by approximately 26 and 50% compared to the control at 24 and 48 h post-treatment (Figure S1A), respectively, bacterial infection into *c-fos* siRNA-treated cells was 0.74 and 0.5-fold that of the control to ensure that the MOI was equivalent for all cells tested.

The internalization assay was performed as previously reported with few modifications (Nichols et al., 1993; de Boer et al., 1996; Pils et al., 2006; Reyes et al., 2017). Briefly, macrophages (10^6^ cells) were seeded in 6-well plates followed by *c-fos* or control siRNA treatment and incubation for 48 h at 37°C in 5% CO_2_. FITC-labeled *B. abortus* at 10^9^ CFU and 0.5 × 10^9^ CFU were used to infect the control and *c-fos* siRNA-treated cells, respectively. After 30 min of infection, the infected cells were collected, washed twice with PBS and subjected to analysis by a FACS Calibur flow cytometer (BD Biosciences, USA). Ethidium bromide (50 μg/ml) was added prior to analysis to quench the green signal produced by outer-membrane-bound FITC-labeled *Brucella*.

The intracellular growth assay was performed as previously reported with few modifications (Pils et al., 2006; Hop et al., 2017b). Briefly, macrophages (10^6^ cells) were seeded in 6-well plates followed by incubation for 24 h and then transfected with *c-fos* or control siRNA. After 24 h of incubation, the control cells were infected with 10^7^ CFU of the virulent *B. abortus*, while the *c-fos* siRNA-treated cells were infected with 0.74 × 10^7^ CFU. After 1 h of infection, RPMI 1640 medium containing 10% (v/v) FBS and gentamycin (30 μg/ml) were added to kill extracellular bacteria, and the infected cells were incubated at 37°C in 5% CO_2_. At 2, 24, and 48 h pi, cells were fixed with 4% paraformaldehyde, permeabilized with 0.1% (v/v) Triton X-100 in PBS and blocked with blocking buffer [2% (v/v) goat serum in PBS]. The cells were stained with rabbit anti-*B. abortus* serum (1:500) followed by secondary incubation with FITC-conjugated anti-rabbit IgG (1:500). The infected cells were collected, washed twice with PBS and subjected to analysis by a FACS Calibur flow cytometer (BD Biosciences). Ethidium bromide (50 μg/ml) was also added prior to analysis to quench the green signal from FITC-conjugated antibody non-specifically bound to the outer membrane.

### Antibiotic assay for bacterial intracellular replication

This assay was performed as previously reported (Kim et al., 2004; Hop et al., 2018). Briefly, RAW 264.7 macrophages (10^6^ cells) were seeded in a 96-well plate, incubated for 24 h at 37°C in 5% CO_2_ and infected with *B. abortus* (10^7^ CFU). After 1 h of infection, the medium of the *B. abortus*-infected macrophages was replaced with RPMI/10% (v/v) FBS and gentamycin (30 μg/ml). At 2, 24, or 48 h post-infection, the cells were washed with PBS, lysed with distilled water and plated on *Brucella* agar.

### RNA isolation and qRT-PCR

Total RNA content was isolated from uninfected or infected RAW 264.7 cells at different time points using a Qiagen RNeasy Kit (Germany). DNA was removed during the extraction using the Qiagen “On-Column DNase Digestion” protocol.

Quantitative real-time PCR analysis was performed as previously described (Gutierrez et al., 2008; Hop et al., 2017b). Briefly, a mixture of SYBR Green PCR Master Mix (Applied Biosystems, USA) and different pairs of 10 pM primers (Tables 1, 2) were denatured at 95°C for 10 min followed by 40 PCR cycles of 95°C for 15 s, 55°C for 30 s, and 60°C for 32 s. The mRNA expression profiles were normalized to β-actin. The fold increase of each gene was calculated using the 2^−ΔΔCT^ method.

**Table 1:** List of primer sequences of cytokines, c-Fos and cyclin family genes used for qRT-PCR.

**Gene**	**Common name**	**Forward primer**	**Reverse primer**
*b-actin*	β-actin	5′-CGCCACCAGTTCGCCATGGA-3′	5′-TACAGCCCGGGGAGCATCGT-3
*Il1b*	Interleukin 1β	5′-CAACCACACAAGTGATATTC-3′	5′-GGATCCACACTCTCCAGCTG-3
*Il6*	Interleukin 6	5′-TCCAGTTGCCTTCTTGGGAC-3′	5′-GTACTCCAGAAGACCAGAG-3′
*Tnf*	Tumor necrosis factor	5′-CACAGAAAGCATGATCCGCG-3′	5′-CGGCAGAGAGGAGGTTGACT-3′
*Il10*	Interleukin 10	5′-TGGCCCAGAAATCAAGGAGC-3′	5′-CAGCAGACTCAATACACACT-3′
*c-fos*	Cellular oncogene Fos	5′-CAGATCTGTCCGTCTCTAGTG-3′	5′-CAGCAGACTGGGTGGGGAGTC-3′
*Ccna1*	Cyclin A1	5′-GAGAAGAACCTGAGAAGCAGG-3′	5′-GGATCCTGGCCAAGCTGAGCT-3′
*Ccna2*	Cyclin A2	5′-CGCTGCATCAGGAAGACCAAG-3′	5′-CCTTAAGAGGAGCAACCCGTC-3′
*Ccnd1*	Cyclin D1	5′-CCGCAAGCATGCACAGACCT-3′	5′-GTGGGTTGGAAATGAACTTC-3′
*Ccnd2*	Cyclin D2	5′-GCTCTGTGCGCTACCGACTT-3′	5′-CACGCTTCCAGTTGCAATCA-3′
*Ccne1*	Cyclin E1	5′-CGGACCACAGCAACATGAAAG-3′	5′-CAGGGCTGACTGCTATCCTCG-3′
*Ccne2*	Cyclin E2	5′-CCAGACTCTCCGCAAGAAACC-3′	5′-GTTGGCCACCTGTACTGTCTGG-3′

**Table 2:** List of primer sequences of trafficking regulators and lysosomal enzymes used for qRT-PCR.

**Gene**	**Common name**	**Forward primer**	**Reverse primer**
*Rab1*	Rab1	5′-CCTTCAATAACGTTAAACAGT-3′	5′-TAGTCTACTACTTTCTTTGTGG-3′
*Rab5a*	Rab5a	5′-GTACTACCGAGGAGCACAAG-3′	5′-AAGCTGTTGTCATCTGCATAG-3′
*Rab5b*	Rab5b	5′-GACTAGCAGAAGTACAGCCAG- 3′	5′-CAATGGTGCTTTCCTGGTATTC-3′
*Rab7*	Rab7	5′-CCTCTAGGAAGAAAGTGTTGC-3′	5′-TTCTTGACCGGCTGTGTCCCA- 3′
*Rab9*	Rab9	5′-GCCCATGCAGATTTGGGACAC-3′	5′-GCCGGCTTGGGCTTCTTCTGTA-3′
*Rab10*	Rab10	5′-GCCGAATGTTACTAGGGAACAAG-3′	5′-GCCGCCTCCTCCACTGCTGATA-3′
*Rab11*	Rab11	5′-GAGCAGTAGG TGCCTTATTGG-3′	5′-GAACTGCCCTGAGATGACGTA-3′
*Rab14*	Rab14	5′-GCCGGAGCTACTATAGAGGAGCT-3′	5′-GCCGTTCTGATAGATTTTCTTGG-3′
*Rab20*	Rab20	5′-CTGCTGCAGCGCTACATGGAGCG- 3′	5′-CTCCGCGGCAGTACAGGGAGC-3′
*Rab22a*	Rab22a	5′-GCCGACAAGAACGATTTCGTGCA-3′	5′-GCCGACTTCTCTGACATCAGTA-3′
*Rab24*	Rab24	5′-GCGCGGGTGAGCACCGCAGGGC-3′	5′-GCCTCAGACCCCAACCCCAAG-3′
*Rab31*	Rab31	5′-GCCCAGAAAACATTGTGATGGCG-3′	5′-GGCATTCTTCGCGCTGGTCTCC-3′
*Rab32*	Rab32	5′-GCCGAGTATACTATAAGGAAGCTC-3′	5′-GCCCTGGGAAGGACTCTGGCTG-3′
*Rab34*	Rab34	5′-GCAAAGTGACCCCGTGTGGCGGG−3′	5′-GGGCGTCCCGAAGACCACTCGG-3′
*Eea1*	Early endosome antigen 1	5′-GCCCAATGAAGAGTCAGCAAGTC-3′	5′-GCCCACCTTGAGATGCTGGCGC-3′
*Rilp*	Rab-interacting lysosomal protein	5′-CAGGAACAGCTACAGCGCCTCCT-3′	5′-CTGAGGTTGCCGCATCAGGTTC-3′
*Lamp1*	Lysosomal membrane glycoprotein 1	5′-GGCCGCTGCTCCTGCTGCTGCTG- 3′	5′-ATATCCTCTTCCAAAAGTAATTG- 3′
*Lamp2*	Lysosomal membrane glycoprotein 2	5′-AGGGTACTTGCCTTTATGCAGAAT-3′	5′-GTGTCGCCTTGTCAGGTACTGC−3′
*Lyz1*	Lysozyme 1	5′-CTCTCCTGACTCTGGGACTCCTCC-3′	5′-CTGAGCTAAACACACCCAGTCAGC-3′
*Lyz2*	Lysozyme 2	5′-GGCCAAGGTCTACAATCGTTGTG−3′	5′-GCAGAGCACTGCAATTGATCCCA−3′
*HexA*	Hexosaminidase A	5′-GCCGGCTGCAGGCTCTGGGTTTC- 3′	5′-GCGCGGCCGAACTGACATGGTAC- 3′
*HexB*	Hexosaminidase B	5′-CCCGGGCTGCTGCTGCTGCAGGC- 3′	5′- GTGGAATTGGGACTGTGGTCGATG- 3′
*Hexdc*	Hexosaminidase D	5′-CCACGCCATTTAAGATGAGATTAG-3′	5′-GGCCCTCAGCAGCCTCAGGTGGCC-3′
*Gla*	Galactosidase, α	5′-GGCCATGAAGCTTTTGAGCAGAG- 3′	5′- AGTCAAGGTTGCACATGAAACGTT- 3′
*Glb1*	Galactosidase, β1	5′-GGAGGTGCAGCGGCTGGCCAGAGC-3′	5′-GGTGACATTATAGATGCCGTGCGC-3′
*Glb1l*	Galactosidase, β1 like	5′-GTGACGGGTGGGAAAGCCCTCACC-3′	5′-CTGTCATGTTCCCGATCCACAACG-3′
*Lpl*	Lipoprotein lipase	5′- CAGACATCGAAAGCAAATTTGCCC-3′	5′- GTCCATCCATGGATCACCACGAAG-3′
*CtsA*	Cathepsin A	5′-GCCCTCCCCGGCCTGGCCAAGCAG-3′	5′-GCCGGCTGGATCAGAAAGGGGCCG-3′
*CtsB*	Cathepsin B	5′-GCCGTGGTGGTCCTTGATCCTTCTT-3′	5′-GCCCCTCACCGAACGCAACCCTTC-3′
*CtsC*	Cathepsin C	5′-GCCGCCACACAGCTATCAGTTACTG-3′	5′-GCCCCTGGAGACCTCCAAGATGTGC-3′
*CtsD*	Cathepsin D	5′-CGTCTTGCTGCTCATTCTCGGCCTC- 3′	5′-CACTGGCTCCGTGGTCTTAGGCGAT- 3′
*CtsE*	Cathepsin E	5′-GGAGCAGAGTGAGAGAGAAGCTAC-3′	5′- GGGCCCGTAGTTTCTTCCGAAGGG-3′
*CtsF*	Cathepsin F	5′-GCC GCA GGC TCC GCC TCG-3′	5′-GCC GCT CCT AGC ACG GCC-3′
*CtsG*	Cathepsin G	5′- CCTGTGCACACCTGTATCTACATAA-3	5′- CTGTGTACCGAGTCACCGTACACGC-3′
*CtsH*	Cathepsin H	5′- CTGAGAACCCTTCTTCCCAAGAGC−3′	5′- AGCAGCCAGGCCCCAGCGCACAGC−3′
*CtsK*	Cathepsin K	5′- GGATGAAATCTCTCGGCGTTTAAT-3′	5′- GTCTCCCAAGTGGTTCATGGCCAG-3′
*CtsL*	Cathepsin L	5′-GCCCCTTTTGGCTGTCCTCTGCTT-3′	5′-GCCCTCCATGGAAAAGCCGTGC-3′
*CtsO*	Cathepsin O	5′-GCCCGCAGTTGGTGAACCTCTTGCT-3′	5′-GCCGTCCTTCTGCTGGGTATCTGGG-3′
*CtsS*	Cathepsin S	5′-GCCGACTACCATTGGGATCTCTGGA-3′	5′-GCCGTCTCCCATATCGTTCATGCCC-3′
*CtsZ*	Cathepsin Z	5′- GGCGTCGTCGGGGTCGGTGCAGCA- 3′	5′- CTGCGCCCCAGCAGAGCCAGCTG- 3′

### F-actin staining

F-actin organization was examined in RAW 264.7 cells by immunofluorescence microscopy as previously described (Lee et al., 2013a; Reyes et al., 2017). Briefly, *c-fos* or control siRNA-treated cells were infected with FITC-conjugated bacteria for 30 min and fixed with 4% paraformaldehyde for 1 h at 37°C. The cells were then permeabilized with 0.1% (v/v) Triton X-100 for 10 min and incubated with blocking buffer [2% (v/v) goat serum in PBS] for 30 min at 37°C in 5% CO_2_. The cells were incubated with 0.1 μM rhodamine-phalloidin for 30 min and washed three times with PBS. F-actin organization was observed by fluorescence microscopy.

### FACS assay for F-actin content evaluation

The relative F-actin content in *B. abortus*-infected and uninfected cells with or without *c-fos* siRNA treatment was evaluated as previously reported (Lee et al., 2013b; Reyes et al., 2017). Briefly, RAW 264.7 cells (10^6^ cell/well) were cultured in 6-well plates and treated with *c-fos* or control siRNA for 48 h prior to infection. The cells were infected for 30 min and fixed with paraformaldehyde for 1 h. The cells were then permeabilized and stained with 20 μg/ml lysophosphatidylcholine containing 1 μM tetramethyl rhodamine isothiocyanate-phalloidin for 1 h at 37°C. The cells were centrifuged at 300 × *g* for 5 min at 4°C and washed three times with PBS. The F-actin content was quantified by FACS analysis using a FACS Calibur flow cytometer (BD Biosciences) and is represented on log-scale histograms depicting 10,000 cells. The average F-actin content of a population was expressed as the mean fluorescence intensity.

### Protein quantification by indirect immunofluorescence and FACS

This assay was performed as previously described (Gutierrez et al., 2008; Hop et al., 2017b). Briefly, cells were fixed with 4% (v/v) paraformaldehyde for 1 h, permeabilized with 0.1% Triton X-100 for 10 min and incubated with blocking buffer [2% (v/v) goat serum in PBS] for 1 h at 37°C. Then, either FITC-rabbit polyclonal anti-TLR4 or phosphor-MAPK antibodies in blocking buffer (1:100) were added and incubated for 1 h at 37°C in the dark. Cells were mounted with Permafluor mounting medium and analyzed by using a laser scanning confocal microscope (Olympus FV1000, Japan), and the images were processed using FV10-ASW Viewer 3.1 software. For the FACS assay, after antibody incubation, cells were washed three times with PBS and subjected to FACS on a FACS Calibur flow cytometer (BD Biosciences). The data are represented on log-scale histograms depicting 10,000 cells.

### Western blot analysis

The cell lysates were analyzed by western blotting as previously described (Hop et al., 2018). Briefly, cell lysates were collected at the indicated time points and boiled in 2 × SDS buffer. Protein samples were separated by electrophoresis on 10% (v/v) SDS-PAGE gels and then transferred to Immobilon-P membranes (EMD Millipore, USA) using a semi-dry electroblot assembly (Bio-Rad, USA). The membranes were blocked with 5% (w/v) skim milk (Difco, USA) and subsequently incubated with primary antibodies (1:2,000) in blocking buffer. After washing with 0.05% (v/v) PBS-T, membranes were incubated with horseradish peroxidase (HRP)-conjugated goat anti-mouse IgG antibody (1:5,000) in blocking buffer. The proteins were detected with ECL solution (Thermo Scientific).

### LAMP-1 CtsA and CtsL colocalization

The colocalization of *Brucella*-containing phagosomes (BCPs) with LAMP-1 CtsA and CtsL was performed as previously reported (Kim et al., 2004; Hop et al., 2018). Briefly, RAW 264.7 cells were treated with *c-fos* or control siRNA prior to infection. After 24 h of infection, the infected cells were fixed with 4% (v/v) paraformaldehyde, permeabilized with 0.1% (v/v) Triton X-100 and blocked with blocking buffer [2% (v/v) goat serum in PBS]. The samples were stained with rat anti-LAMP-1, anti-CtsA and anti-CtsL in blocking buffer (1:100) followed by secondary incubation with Texas red-goat anti-rat IgG (1:500). Intracellular *B. abortus* was identified by staining with anti-*B. abortus* rabbit serum (1:100) and FITC-conjugated anti-rabbit IgG (1:100). Colocalization was observed by a laser scanning confocal microscope (Olympus FV1000), and the images were processed using FV10-ASW Viewer 3.1 software. The colocalization percentage of these proteins with the BCPs was determined for 100 randomly selected cells.

### FACS assay for apoptosis and necrosis

The apoptosis and necrosis were evaluated by flow cytometry using apoptosis and necrosis detection kit (Abcam, Cambridge, USA) at 48 h post-infection (pi) in accordance with the manufacturer's instructions. Briefly, after 2 days incubation at 37°C, infected macrophages were stained with apoptosis indicator (apopxin green) and necrosis indicator (7-AAD). The apoptosis and necrosis percentages were determined from 10,000 random cells.

### Cytokine quantitation

The levels of TNF, IL-6, IL-10, and IL-1β in culture supernatants were determined by sandwich ELISA in accordance with the manufacturer's instructions (R&D Systems and Thermo Fisher Scientific, USA).

### Statistical analysis

The data are expressed as the mean ± standard deviation (SD). ANOVA with Tukey's HSD exact test was used to statistically compare the groups. The results with *P* < 0.05 were considered significantly different.

## Results

### c-Fos controls cell proliferation and bacterial infection via two distinct mechanisms in *B. abortus*-infected macrophages

**Figure 1:**
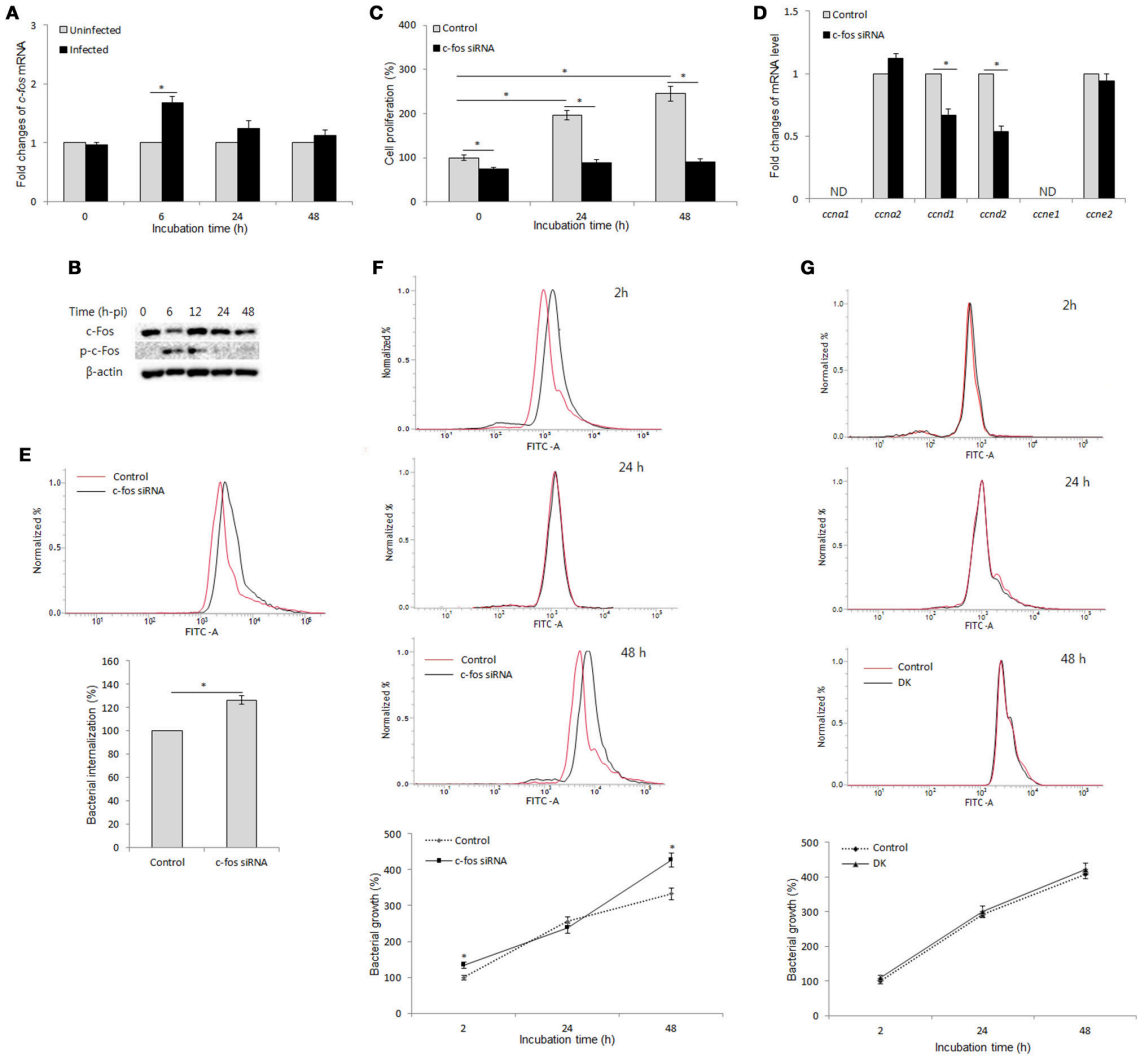
c-Fos controls cell proliferation and bacterial infection via two distinct mechanisms in *B. abortus*-infected macrophages. **(A)** RAW 264.7 cells were infected with *B. abortus*, and the transcriptional profiles of *c-fos* were examined by qRT-PCR. **(B)** The activation of c-Fos in *B. abortus*-infected cells was evaluated by western blotting at different time points. **(C)** Cells were treated with *c-fos* siRNA prior to *B. abortus* infection, and cell proliferation was examined. **(D)** The transcriptional profiles of proliferative genes were assessed by qRT-PCR at 24 h pi. **(E)** Flow cytometry histograms and quantitative analysis of *B. abortus* internalization in *c-fos* or control siRNA-treated cells at 30 min pi. **(F)** Flow cytometry histograms and quantitative analysis of intracellular *B. abortus* growth in *c-fos* or control siRNA-treated cells at the indicated time points. **(G)** Flow cytometry histograms and quantitative analysis of intracellular *B. abortus* growth in *ccnd1/ccnd2* or control siRNA-treated cells at the indicated time points. The data represent the mean ± SD of triplicate experiments. The asterisk indicates significant difference (*P* < 0.05). Abbreviations: ND, not detectable; DK, double knockdown.

To examine the interaction between *B. abortus* infection and c-Fos signaling in macrophages, we first evaluated the effect of *B. abortus* infection on the expression of c-Fos by qRT-PCR and western blot assays at different time points. As shown in Figure 1A, *B. abortus* slightly induced the *c-fos* transcript, by approximately 1.7-fold, during early infection; however, the activation of c-Fos was stimulated by *B. abortus* as early as 6 h post-infection (pi) and was stable until 12 h post-infection (pi) (Figure 1B).

On the other hand, because cell proliferation has many roles in host immunity and cell cycle control is one of the better-known functions of the c-Fos protein (Pai and Bird, 1994; Galea et al., 1999; Wang et al., 2015), we examined this function in *B. abortus*-infected macrophages. For this investigation, we treated RAW 264.7 cells with *c-fos* siRNA and incubated the treated cells for 1 day prior to *B. abortus* infection, and cell proliferation was evaluated at 0, 24, and 48 h pi. Interestingly, we found that *B. abortus*-infected macrophages still proliferate at 24 h pi but not at 48 h pi, and this phenomenon was shown to be partially dependent on c-Fos signaling (Figure 1C). To complement this result, we assessed the role of c-Fos in the expression of cyclins D1, D2, A1, A2, E1, and E2 (*ccnd1, ccnd2, ccna1, ccna2, ccne1*, and *ccne2*) by qRT-PCR at 24 h pi. As expected, suppression of c-Fos signaling drastically reduced the expression of *ccnd1* and *ccnd2* transcripts but not of *ccna2* and *ccne2* transcripts, whereas *ccna1* and *ccne1* transcripts were not detectable (Figure 1D). These results suggested that c-Fos signaling plays an important role in cell division by controlling two proliferation inducers, CCND1 and CCND2, in the context of *Brucella* infection.

We next treated the cells with *c-fos* siRNA prior to *B. abortus* infection to assess the role of c-Fos in the host response to *Brucella* infection. Due to the regulation of proliferation by c-Fos, we utilized flow cytometry (FACS) rather than the traditional gentamycin assay to evaluate bacterial internalization and intracellular replication. As previously described, labeling with FITC did not affect the infectivity of *B. abortus* (de Boer et al., 1996; Reyes et al., 2017). Furthermore, because live cells uptake ethidium bromide after only 10–15 min of exposure, the addition of ethidium bromide at 50 μg/ml prior to analysis enabled total quenching of the extracellular FITC signal of adherent *Brucella* without influencing the fluorescence of intracellular bacteria (16). Intriguingly, suppression of c-Fos signaling by *c-fos* siRNA was shown to markedly increase invasion by and persistence of *B. abortus* in macrophages (Figures 1E,F), indicating that c-Fos is an important regulator of the innate immune response to *B. abortus* infection. Taken together, these data suggest that c-Fos is important not only for proper cell division during *Brucella* infection but also for efficient restriction of *Brucella* invasion and survival in macrophages.

On the other hand, we treated cells with *Tnf* siRNA and subjected the treated cells to FACS and the traditional gentamycin assay. The suppression of TNF signaling by *Tnf* siRNA treatment was seen to markedly increase bacterial survival during late infection without affecting bacterial internalization (Hop et al., 2017b). As expected, treatment with *Tnf* siRNA resulted in a marked increase in fluorescent intensity at 48 h pi but not during early infection compared to the control (Figure S1B), which is consistent with the results obtained from the gentamycin assay (Figure S1C). Because the trend of infection and the TNF effect were shown to be similar in both of the assays, that FACS assay could be applicable for internalization and intracellular growth evaluation in *c-fos* siRNA-treated cells. Thus, the above findings represent the effect of the c-Fos pathway on *Brucella* infection.

A number of studies have reported that after infection with *Brucella*, macrophages stop proliferating and shifting the cell state to one associated with antibacterial immunity (Eskra et al., 2003; Cha et al., 2013); however, the actual role of this phenomenon in *Brucella* infection remains unknown. Thus, we hypothesized that antimicrobial activity could be associated with proliferative activity during *Brucella* infection. To test this hypothesis, we treated cells with double *ccnd1/ccnd2* siRNAs and evaluated the effect of these siRNAs on *Brucella* persistence in RAW 264.7 cells. As expected, the double knockdown of *ccnd1/ccnd2* genes markedly inhibited cell proliferation during *Brucella* infection (data not shown); however, the double knockdown did not alter bacterial survival (Figure 1G). These data suggest that the proliferative activity is not employed in host defense against *Brucella* infection in macrophages. Thus, our results are indirect evidence of proliferative and antimicrobial regulation by c-Fos via two distinct signaling mechanisms in *Brucella*-infected macrophages.

### Inhibitory role of c-Fos on bacterial uptake via modulation of F-actin polymerization and phagocytic signaling

**Figure 2:**
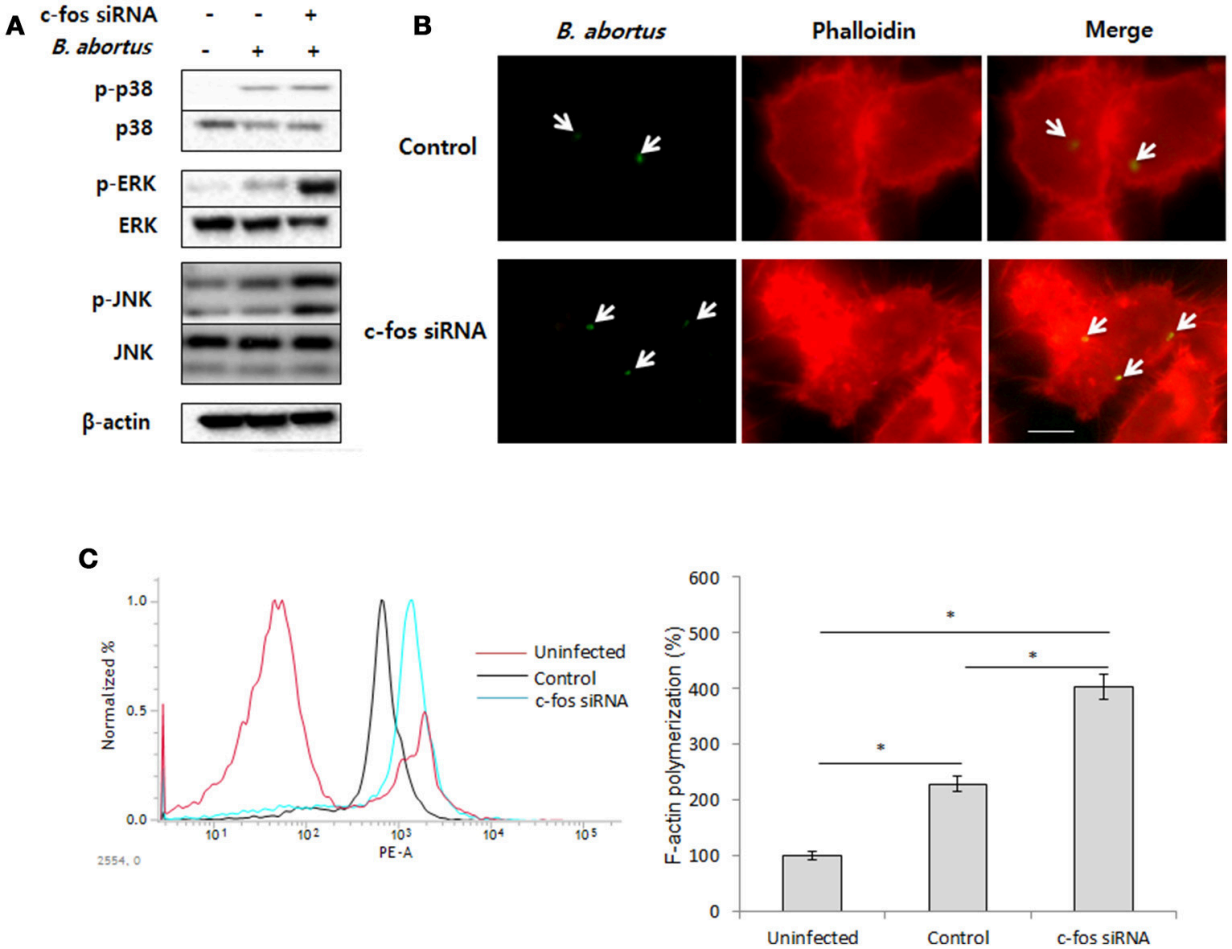
c-Fos inhibits bacterial uptake by modulating F-actin polymerization and phagocytic signaling. RAW 264.7 cells were treated with *c-fos* siRNA prior to *B. abortus* infection. **(A)** MAPK activation was assessed by western blotting at 30 min pi. **(B)** F-actin polymerization was observed by fluorescence microscopy at 30 min pi. **(C)** Flow cytometry histograms and quantitative analysis of F-actin content at 30 min pi. The data represent the mean ± SD of triplicate experiments. The asterisk indicates significant difference (*P* < 0.05). Scale bars=1 μm.

A variety of studies have proven that F-actin polymerization is an essential event for phagocytic uptake of microbial pathogens in both epithelial cells and macrophages (Gruenheid and Finlay, 2003; Lee et al., 2013b). Furthermore, mitogen-activated protein kinase (MAPK) was also clearly shown to play importante roles in the phagocytosis of bacteria and in remodeling of the actin cytoskeleton (Schorey and Cooper, 2003; Doyle et al., 2004). Therefore, we hypothesized that the regulatory role of c-Fos in *Brucella* invasion is associated with the control of MAPK activation, which subsequently alters F-actin polymerization. To test this hypothesis, macrophages were treated with *c-fos* siRNA prior to infection with *B. abortus*. The activation of ERK1/2, p38a, and JNK were first verified by a western blot assay at 30 min pi. Interestingly, we found that the phosphorylation levels of ERK1/2 and JNK but not p38 in c-Fos-suppressing macrophages were markedly increased compared to the infected control cells (Figure 2A). To complement this data, cells subjected to the same treatment were labeled with FITC and assayed by FACS. As expected, the fluorescence of p-ERK1/2 and p-JNK was significantly enhanced, by approximately 31 and 26%, respectively, when the c-Fos signaling was inhibited (Figure S2A). In addition, F-actin polymerization was also assessed at 30 min pi by fluorescence microscopy and FACS assay. Consistent with MAPK activation, increased F-actin polymerization was observed in c-Fos-deficient cells compared to control cells (Figure 2B). In addition, quantitation of F-actin content during *B. abortus* invasion also consistently showed that *c-fos* siRNA pretreatment led to a marked increase in F-actin fluorescence intensity compared with the infected control cells (Figure 2C). Taken together, our findings clearly indicate that inhibition of c-Fos signaling stimulates JNK and ERK1/2 activation, which results in increased F-actin polymerization, finally leading to the enhancement of bacterial uptake by macrophages.

### c-Fos mediates TLR-4 signaling to control *B. abortus* phagocytosis but not intracellular replication

**Figure 3:**
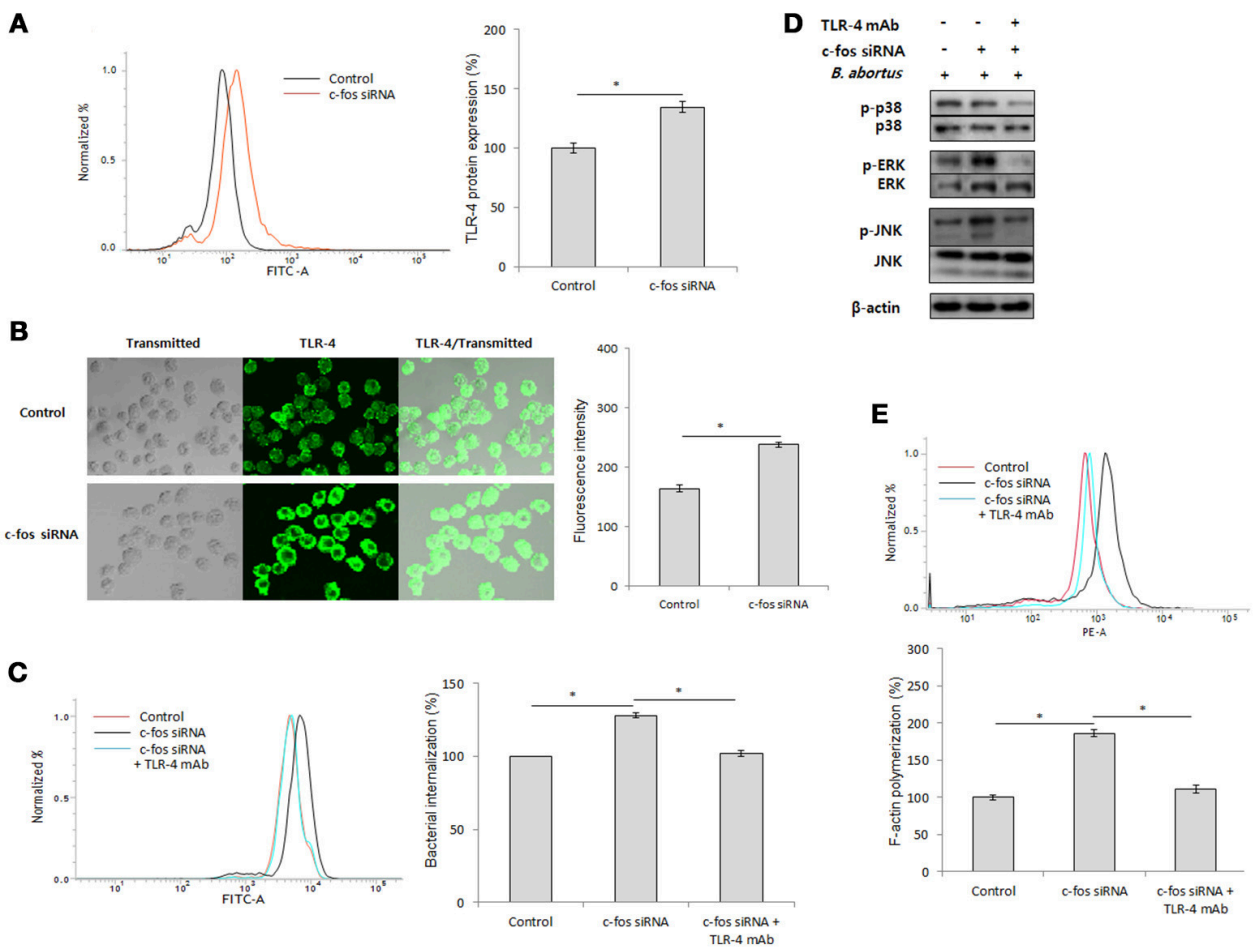
c-Fos mediates TLR-4 signaling to control *B. abortus* phagocytosis but not intracellular replication. **(A)** Flow cytometry histogram and quantitative analysis of TLR-4 expression after 2 days of *c-fos* or control siRNA treatment in RAW 264.7 cells. **(B)** The expression of TLR-4 was confirmed by fluorescence microscopy and its quantitative analysis in *c-fos* or control siRNA-treated cells. **(C)** Flow cytometry histograms and quantitative analysis of *B. abortus* internalization in cells concomitantly treated with *c-fos* siRNA and anti-TLR-4 mAb at 30 min pi. **(D)** The activation of MAPK in cells concomitantly treated with *c-fos* siRNA and anti-TLR-4 mAb was evaluated by western blotting at 30 min pi. **(E)** Flow cytometry histograms and quantitative analysis of F-actin content in cells concomitantly treated with *c-fos* siRNA and anti-TLR-4 mAb at 30 min pi. The data represent the mean ± SD of triplicate experiments. The asterisk indicates significant difference (*P* < 0.05).

As previously reported, Toll-like receptor 4 (TLR4) is a part of the immune system that contributes to *B. abortus* internalization in macrophages by promoting phagocytic signaling, including MAPK activation and F-actin polymerization (Lee et al., 2013b). In addition, c-Fos has been reported to be activated by TLR-4 signaling (Liu et al., 2009; Wang et al., 2015), suggesting the potential involvement of TLR-4 in the immune regulation of c-Fos signaling in the context of *Brucella* infection. To test this hypothesis, we first treated the cells with c-Fos siRNA and verified the expression of the TLR-4 protein by FACS. Interestingly, in uninfected RAW 264.7 cells, inhibition of c-Fos signaling resulted in a significant increase in TLR-4 protein expression (Figure 3A). Consistent with this finding, further validation by indirect immunofluorescence also showed increased TLR-4 levels in c-Fos-deficient cells compared with the control (Figure 3B), indicating that c-Fos is involved in looped regulation of TLR-4 in RAW 264.7 cells. To determine the actual role of TLR-4 in the c-Fos-modulated immune response to *Brucella* infection, we concomitantly treated RAW 264.7 cells with *c-fos* siRNA and anti-TLR-4 mAb. Invasion and survival of *B. abortus* were then evaluated by FACS. Interestingly, inhibition of TLR-4 by mAb was found to significantly reduce bacterial uptake (Figure 3C); however, TLR-4 inhibition did not increase the bacterial killing capacity of c-Fos-deficient macrophages (Figure S2B). Furthermore, evaluation of key effectors of B. abortus phagocytosis, including MAPK activation (Figure 3D) and F-actin polymerization (Figure 3E), showed that these effectors were also suppressed when TLR-4 signaling was inhibited in c-Fos-inhibiting cells. Therefore, these findings indicate that TLR-4 is a major downstream molecule of c-Fos signaling that plays an important role in *Brucella* phagocytosis but not anti-*Brucella* immunity in murine macrophages.

### c-Fos has both pro- and anti-inflammatory effects during *B. abortus* infection in RAW264.7 cells

**Figure 4:**
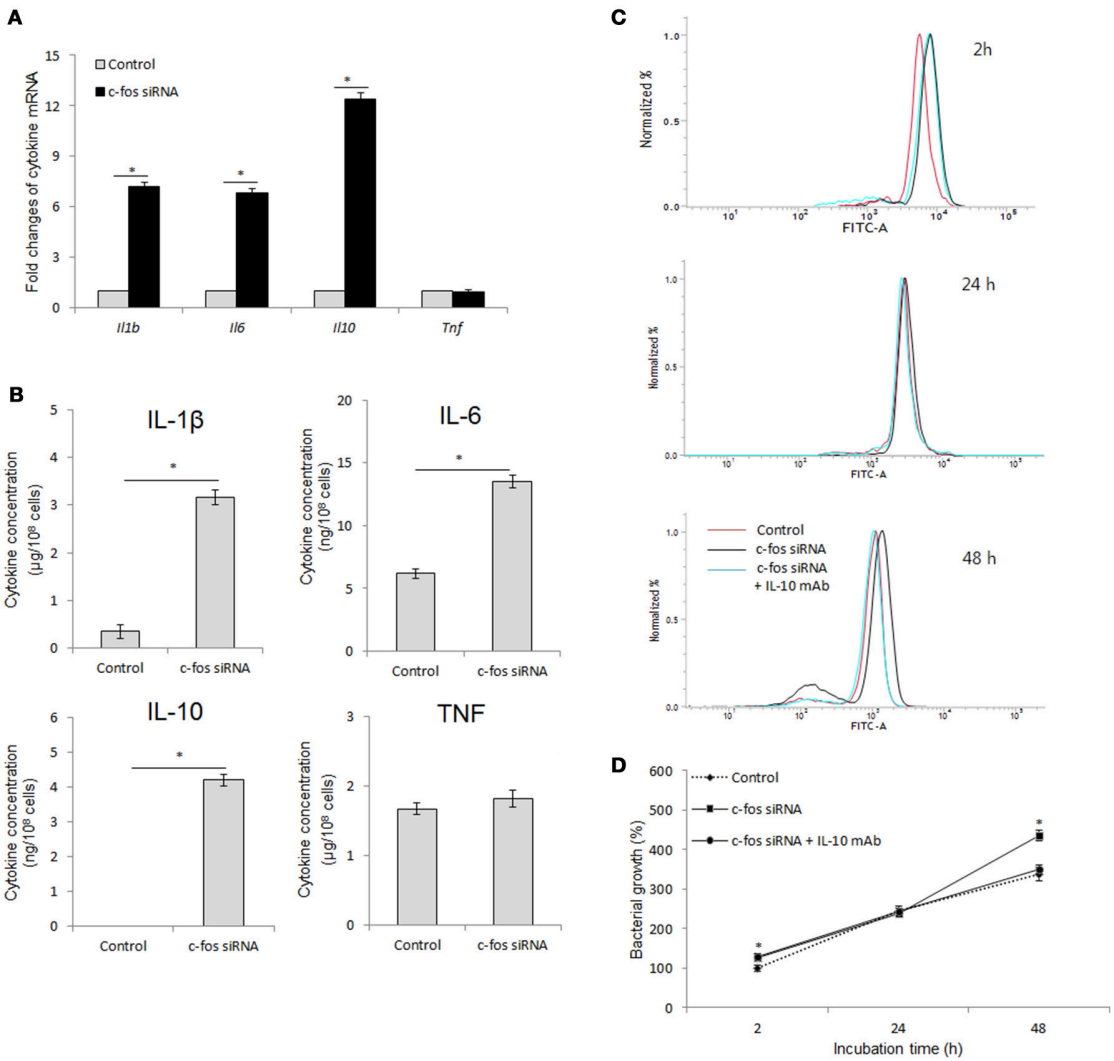
c-Fos has both pro- and anti-inflammatory effects during *B. abortus* infection in RAW264.7 cells. **(A)** Cells were pretreated with *c-fos* siRNA before *B. abortus* infection, and total RNA was isolated. The transcriptional profiles of cytokine genes were evaluated by qRT-PCR. **(B)** Cytokine production was evaluated by sandwich ELISA. **(C)** Flow cytometry histograms and **(D)** Quantitative analysis of intracellular *B. abortus* growth in cells concomitantly treated with *c-fos* siRNA and anti-IL-10 mAb. The data represent the mean ± SD of triplicate experiments. The asterisk indicates significant difference (*P* < 0.05).

Inflammation-related cytokines have been shown to be associated with host resistance to *Brucella* infection (Jiang and Baldwin, 1993; Xavier et al., 2013; Hop et al., 2017b). Moreover, another study by Ray et al. (2006) also proved the regulatory role of c-Fos in cell inflammation. Thus, we hypothesized that c-Fos controls *Brucella* infection by regulating inflammatory cytokine function. To address this hypothesis, we assessed the expression and secretion of the most important inflammatory cytokines upon treatment with target *c-fos* siRNA. Surprisingly, inhibition of c-Fos signaling resulted in induction of both pro-inflammatory (IL-6 and IL-1β) and anti-inflammatory (IL-10) cytokines (Figures 4A,B), suggesting that c-Fos has both pro- and anti-inflammatory effects during *B. abortus* infection. However, TNF signaling was shown to be independent of c-Fos regulation.

IL-10 is most well known as an anti-*Brucella* immune suppressor (Fernandes and Baldwin, 1995; Xavier et al., 2013; Hop et al., 2018) that is also associated with the TNF pathway (Hop et al., 2017b). In addition, mice lacking any of the direct coordinators of the IL-1B pathway, such as inflammasome NLRP3, IL-1R, MyD88 or IL-18, were shown to be more susceptible to *B. abortus* (Gomes et al., 2013), and our unpublished data indicated that IL-6 signaling is required for proper functioning of several lysosomal enzymes in macrophages until 24 h post-infection, which is important for bacterial survival during the late stage of infection. Thus, these observation indicated that IL-10 may be a major effector of antimicrobial suppression in c-Fos-deficient cells. To test this hypothesis, we concomitantly treated cells with *c-fos* siRNA and anti-IL-10 mAb and then assessed bacterial survival. As shown in Figures 4C,D, inhibition of IL-10 by the target mAb almost restored the bacterial killing in c-Fos-suppressed cells. Taken together, these results clearly show that suppression of c-Fos stimulates the predominant IL-10, which results in an increased survival of intracellular *B. abortus* in cultured macrophages.

### BCPs fail to recruit lysosomal enzymes in c-Fos-deficient RAW 264.7 cells

**Figure 5:**
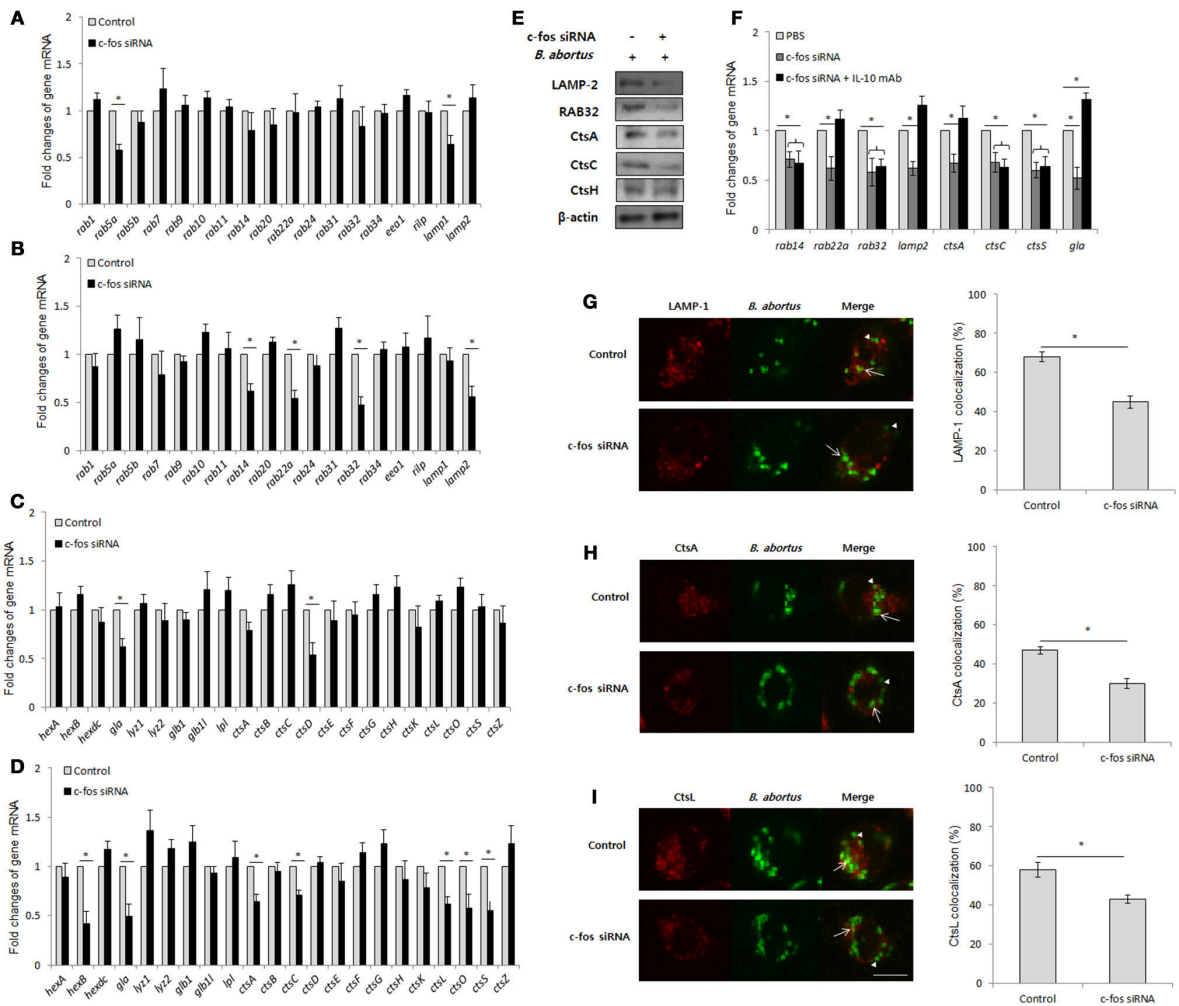
BCPs fail to recruit lysosomal enzymes in c-Fos-deficient RAW 264.7 cells. Macrophages were treated with *c-fos* siRNA prior to *B. abortus* infection. **(A)** Transcriptional profiles of trafficking regulators were evaluated by qRT-PCR at 2 h pi. **(B)** Transcriptional profiles of trafficking regulators were evaluated by qRT-PCR at 24 h pi **(C)** Transcriptional profiles of lysosomal enzymes were evaluated by qRT-PCR at 2 h pi. **(D)** Transcriptional profiles of lysosomal enzymes were evaluated by qRT-PCR at 24 h pi. **(E)** The expression of representatives was evaluated by western blotting at 24 h pi. **(F)** Transcriptional profiles of phagolysosomal genes in cells concomitantly treated with *c-fos* siRNA and anti-IL-10 mAb were assessed by qRT-PCR at 24 h pi. **(G)** The colocalization of BCPs with LAMP-1 was analyzed at 2 h pi. **(H)** The colocalization of BCPs with CtsA and **(I)** CtsL was analyzed at 24 h pi. Arrow, marker positive; arrow heads, marker negative. The percentage of markers colocalized with BCPs in 100 cells was determined. The data represent the mean ± SD of triplicate experiments. The asterisk indicates significant difference (*P* < 0.05).

IL-10 has been previously determined to be an inhibitor of the lysosome-mediated killing process in primary and cultured macrophages (Xavier et al., 2013; Hop et al., 2018), suggesting that c-Fos might also play a regulatory role in phagolysosome fusion during *Brucella* infection. To address this hypothesis, we suppressed c-Fos signaling by target siRNA treatment and subsequently evaluated the transcriptional profiles of a variety of phagolysosome-related genes by qRT-PCR at 4 and 24 h pi. Interestingly, during early infection, inhibition of c-Fos signaling caused the reduction in the expression levels of a few important trafficking regulators and hydrolytic enzymes, including *Lamp1, Rab5a, CtsD* and *Gla* (Figures 5A,C); however, interference of c-Fos signaling was discovered to have greater influence on phagolysosome-related gene expression at 24 h post-infection, with reduction in the levels of 11 transcripts, namely, *Lamp2, Rab14, Rab22a, Rab32, Gla, HexB, CtsA, CtsC, CtsL, CtsO*, and *CtsS*, compared with control cells (Figures 5B,D). Furthermore, western blotting to test for the expression of 5 selected proteins, namely, LAMP-2, Rab32, CtsA, CtsC, and CtsH, was carried out at 24 h pi. Our data revealed that the levels of all the proteins except CtsH were reduced when c-Fos signaling was inhibited (Figure 5E), suggesting a regulatory role for the c-Fos protein in the expression of different trafficking regulators and lysosomal enzymes.

In addition, to determine the contribution of IL-10 on this effect, we concomitantly suppressed the c-Fos and IL-10 signaling pathways by *c-fos* siRNA and IL-10 mAb, respectively, and evaluated the expression of 8 c-Fos-modulated representatives by qRT-PCR. Intriguingly, suppressing IL-10 signaling only restored the levels of 4 transcripts, namely, *Lamp2, Rab22a, Gla*, and *CtsA*, while the other 4 genes (*Rab14, Rab32, CtsC*, and *CtsS)* were seen to be independent of IL-10 modulation (Figure 5F). These data indicate that c-Fos controls the expression of phagolysosomal genes via IL-10-dependent and independent mechanisms.

Since the recruitment of lysosomal enzymes by *Brucella*-containing phagosomes (BCP) is required for efficient killing of intracellular bacteria, and the inhibition of expression of several phagolysosomal genes was observed in c-Fos-deficient infected cells, the delivery of lysosomal enzymes to the *Brucella* phagosome might also be insufficient when c-Fos signaling is inhibited. To test this hypothesis, we evaluated the fraction of BCP that could be labeled for the LAMP-1 (2 h pi), CtsA or CtsL (24 h pi) proteins. As expected, the colocalization of BCPs with LAMP-1 (Figure 5G), CtsA (Figure 5H) and CtsL (Figure 5I) was markedly reduced in c-Fos-inhibited cells relative to the controls, suggesting that c-Fos controls the recruitment of these markers by BCPs. In summary, these findings describe for the first time the regulatory role of c-Fos in lysosome-mediated killing of *B. abortus* in murine macrophages.

### The c-Fos/trail pathway controls the outcome of *B. abortus* infection by governing cell necrosis during late infection

**Figure 6:**
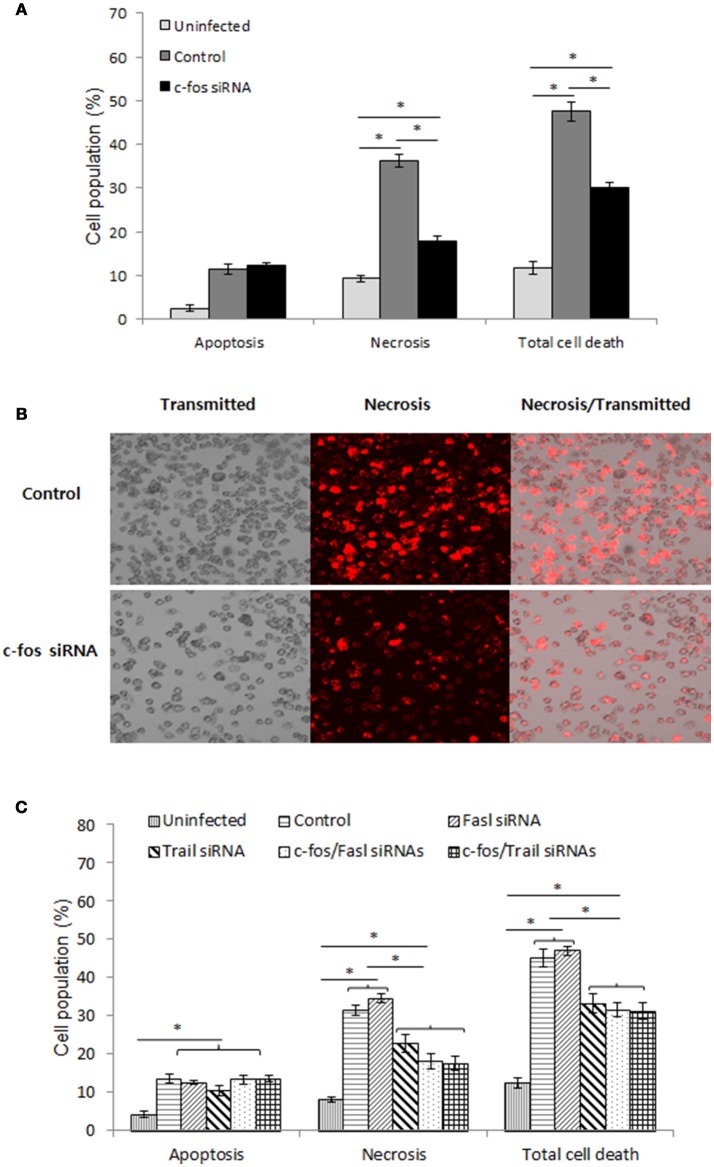
The c-Fos/TRAIL pathway controls the outcome of *B. abortus* infection by governing cell necrosis during late infection. RAW 264.7 macrophages were treated with different siRNAs prior to *B. abortus* infection and subjected to staining with apopxin green (apoptosis) or 7-AAD (necrosis). **(A)** Quantitative analysis of the flow cytometry assay for apoptosis and necrosis from 10,000 events at 48 h pi. **(B)** Cell necrosis was observed by fluorescence microscopy at 48 h pi. **(C)** Quantitative analysis of the flow cytometry assay for apoptosis and necrosis from 10,000 events at 48 h pi. The data represent the mean ± SD of triplicate experiments. The asterisk indicates significant difference (*P* < 0.05).

As recently hypothesized, after reaching the ER-like compartment, in addition to replication, *Brucella* was also reported to self-dissociate, leading to induction of cell death by apoptosis and necrosis (Turse et al., 2011; Pei et al., 2014). This phenomenon is thought to be important for bacterial egress and dissemination; however, elucidation of the underlying mechanism of this process requires further investigation. Moreover, c-Fos signaling has been reported to induce apoptosis in different cell types (Preston et al., 1996; Siegmund et al., 2001; Zhang et al., 2007), suggesting that c-Fos might also play this role during *Brucella* infection. To test this hypothesis, we treated the cells with c-Fos siRNA prior to *Brucella* infection. After 2 days of infection, cells were stained with apoptotic and necrotic dyes and subjected to FACS analysis. Consistent with our previous observation (Hop et al., 2017b), *B. abortus* infection caused approximately 28% and 10% cell necrosis and apoptosis, respectively; however, interestingly, we found that suppression of the c-Fos pathway significantly reduced, by approximately 14%, necrosis but not apoptosis in these cells (Figure 6A, Figure S3A). To complement this data, we stained the cells with necrotic dye only and observed the cells by fluorescence microscopy. As expected, inhibition of c-Fos signaling significantly reduced cell necrosis (Figure 6B), indicating the importance of c-Fos in *B. abortus*-induced host-cell death.

To identify the molecules that contribute to this function of c-Fos, we treated cells with either Fasl, Trail, c-Fos, c-Fos/Fasl or c-Fos/Trail siRNAs and infected the cells with *B. abortus*. Two days after infection, FACS was carried out, and the results revealed that, in addition to c-Fos siRNA, treatment with Trail siRNA also reduced necrosis in infected cells by approximately 13%. In addition, double knockdown of c-Fos/Trail had a similar reduction rate to single c-Fos or Trail siRNA treatment, suggesting that c-Fos and TRAIL function in similar pathways that play important roles in the regulation of cell necrosis during *Brucella* infection, whereas interference of FasL signaling does not influence the cell death process (Figure 6C, Figure S3B). Taken together, our data clearly indicated that the c-Fos/TRAIL pathway is required for necrosis in *B. abortus*-infected macrophages.

## Discussion

*B. abortus* is a gram-negative bacterium that can invade and proliferate within macrophages. Hence, this bacterium inhibits essential host-defense mechanisms of killing that are still not well understood (Hop et al., 2018). There is a general agreement that inflammation is one of the most important responses against microbial pathogens, and in the context of *Brucella* infection, inflammation has been shown to play important roles in host resistance, especially in the brucellacidal activity of macrophages (Jiang and Baldwin, 1993; Hop et al., 2017b), suggesting that master inflammatory regulators such as nuclear factor kappa-light-chain-enhancer of activated B cells (NF-*k*B) and AP-1 could potentially contribute to host immunity during *Brucella* infection. Thus, in this study, we functionally characterized c-Fos, a component of AP-1 in *B. abortus*-infected macrophages, which could provide new insights into macrophage-*Brucella* interactions.

In this study, we report for the first time the requirement of c-Fos signaling for efficient restriction of *B. abortus* phagocytosis by macrophages. The suppression of c-Fos by target siRNA significantly increased *Brucella* internalization, which occurred in conjunction with elevated activation of ERK1/2 and JNK as well as enhanced F-actin polymerization. TLR-4 has previously been shown to be an important initiator of c-Fos signaling when cells are triggered by lipopolysaccharide (LPS) or viral infection (Liu et al., 2009; Wang et al., 2015). However, we present here the first evidence of negative-feedback-looped regulation by c-Fos of the TLR-4 pathway as well as the contribution of TLR-4 to regulatory functions of bacterial c-Fos in macrophages. Inhibition of c-Fos signaling resulted in a marked induction of TLR-4 in unstimulated cells, which subsequently stimulated MAPK activation and F-actin polymerization and enhanced *Brucella* internalization. Our data also reconfirmed the role played by TLR-4 in *B. abortus* infection as previously observed (Lee et al., 2013b); however, the role of TLR-4 is now known to be regulated by c-Fos signaling. These data suggest an important role for c-Fos in infection by other bacteria, including *Mycobacterium tuberculosis* and *Salmonella typhimurium*, which have also been shown to be controlled by TLR-4 (Abel et al., 2002; Arpaia et al., 2011).

Previously, transcriptomic studies have shown that *Brucella* infection does not induce expression of *c-fos* during late infection (Eskra et al., 2003; Cha et al., 2013; Hop et al., 2017a); therefore, little attention has been given to the immunological role of c-Fos during studies of Brucella pathogenesis. However, interestingly, we found that although the expression of c-fos was transiently induced by B. abortus during early infection, its activation until 12 h pi is required for the subsequent activation of an efficient Brucella clearance. Suppression of c-Fos by target siRNA markedly increased the number of bacteria within macrophages in conjunction with pro-inflammatory changes. Surprisingly, we found that c-Fos deficiency resulted in the induction of both anti-inflammatory (IL-10) and pro-inflammatory (IL-6 and IL-1β) cytokines during *Brucella* infection, whereas TNF signaling was shown to be independent of c-Fos. These findings seemed to be contrary to previous investigations, in which c-Fos was indicated to play the role of a pro-inflammatory suppressor of macrophages (Ray et al., 2006); however, we could not explain how c-Fos inhibits both anti- and pro-inflammatory pathways during *B. abortus* infection in the current study.

In previous reports, IL-10 was clearly seen to promote *Brucella* persistence in host cells by suppressing lysosome-mediated killing (Fernandes and Baldwin, 1995; Xavier et al., 2013), while TNF was recently proven to mediate NF-*k*B to control *Brucella*-killing effectors such as reactive oxygen species (ROS) and nitric oxide (NO) (Hop et al., 2017b). In addition, although the function of IL-1β in the immune response to *Brucella* infection has not been reported, the anti-*Brucella* effects of direct coordinators of the IL-1β pathway, such as inflammasome NLRP3, IL-1R, MyD88 and IL-18, have been clearly demonstrated (Gomes et al., 2013), suggesting that IL-1β likely contributes to the *Brucella*-killing activity. Moreover, our unpublished data proved that IL-6 signaling is required for the efficient killing of *B. abortus in vitro* and *in vivo*. Therefore, in this study, the marked increase of bacterial replication in c-Fos-deficient cells was hypothesized to mainly depend on elevated IL-10 production. Our data in turn have proved this hypothesis, as concomitant suppression of the IL-10 pathway by IL-10 mAb significantly restored bacterial killing in c-Fos-deficient cells. These findings suggest that c-Fos can regulate both anti- and pro-inflammatory states in macrophages; however, in the presence of *B. abortus*, the predominant role of c-Fos signaling is inhibition of anti-inflammatory cytokine IL-10 in order to induce pro-inflammatory antibacterial response in cells.

Moreover, the implication of TLR-4 in the anti-*Brucella* activity of c-Fos was also assessed in the current study; however, our evidence showed that TLR-4 plays no role in the downstream brucellacidal activity of c-Fos signaling. Similarly, although the anti-*Brucella* effect of c-Fos is solely dependent on the suppression of IL-10 activity, the function of other pro-inflammatory cytokines (IL-6 and IL-1β) in c-Fos signaling requires further investigation.

Consistent with previous reports on the regulatory role of c-Fos in the cell cycle (Pai and Bird, 1994; Wang et al., 2015), treatment with *c-fos* siRNA was shown to inhibit the proliferation of both normal and *Brucella*-infected RAW 264.7 cells. Our data clearly showed that RAW cells were able to proliferate until 24 h pi, while the cells were previously thought to be inhibited immediately after *Brucella* infection (Eskra et al., 2003). Moreover, we also found that this effect was solely associated with the functional participation of two proliferative executioners, CCND1 and CCND2, since reduction of cell number was accompanied by a marked decrease in *ccnd1* and *ccnd2* transcript levels in conditions of c-Fos deficiency. However, suppression of cell proliferation by a double knockdown of *ccnd1/ccnd2* did not affect *Brucella* invasion and survival, indicating that proliferation and bacterial killing occur independently during *B. abortus* infection. Thus, our data suggest that c-Fos regulates cell proliferation and immune response via two distinct signaling mechanisms under conditions of *Brucella* infection.

One of the striking findings of this study is that c-Fos is required for the sufficient expression and timely recruitment of different trafficking regulators, such as LAMP-1, LAMP-2, Rab5a, Rab14, Rab22a, and Rab32, during *B. abortus* infection. To our knowledge, it has not been previously reported that c-Fos is required for phagolysosome fusion during bacterial infection. Phagolysosome fusion is one of the most important effectors restricting *Brucella* infection in macrophages (Kim et al., 2004; Starr et al., 2008). Although the trafficking of *Brucella*-containing phagosomes (BCPs) in macrophages is regulated by multiple trafficking regulators, little is known about the actual roles of these regulators in this process and in host response and bacterial replication. To date, LAMP-1 is the most well-known regulator that governs the phagolysosome fusion process. The timely recruitment and dissociation of LAMP-1 to BCPs during early infection has been proven to be critical for the subsequent lysosome-mediated killing activity that mainly contributes to the final outcome of *Brucella* infection (Celli et al., 2003; Starr et al., 2008). In addition to LAMP-1, other regulators could also potentially contribute to regulate the fusion; Rab5A and Rab22A have been found to be associated with the stimulation of the maturation of phagosomes containing either *Listeria* or *Mycobacteria* during early infection (Gutierrez, 2013). Similarly, LAMP2 was demonstrated to have overlapping functions with LAMP-1 in the recruitment of Rab7, moving toward the microtubule-organizing center and subsequently fusing with lysosomes (Huynh et al., 2007). In general, our findings suggest that c-Fos could control phagolysosome fusion by regulating different important trafficking regulators.

Moreover, expression of numerous hydrolytic enzymes, including HexB, Gla, CtsA, CtsC, CtsD, CtsO, CtsL, and CtsS, and fractions of BCPs that are labeled by CtsA and CtsL were found to be drastically reduced in c-Fos-deficient infected cells. Several members of the cathepsin family, including CtsB, CtsD, CtsG, CtsO, CtsL, and CtsS, have been shown to contribute to killing intracellular *M. tuberculosis* (Rivera-Marrero et al., 2004; Pires et al., 2016), *M. bovis* (Soualhine et al., 2007) and *S. pneumoniae* (Bewley et al., 2011). Similarly, HexB was also proven to protect macrophages against *Mycobacterium marinum* (Koo et al., 2008), suggesting that c-Fos signaling is required for lysosome-mediated killing activity in *B. abortus*-infected macrophages. Furthermore, because IL-10 was shown to be a major mediator of c-Fos-activated anti-*Brucella* immune responses and because IL-10 was previously demonstrated to suppress phagolysosome fusion in cultured and primary macrophages (Xavier et al., 2013; Hop et al., 2018), c-Fos was hypothesized to be totally dependent on the IL-10 pathway for controlling lysosome-mediated killing. However, surprisingly, we found that IL-10 only partially contributes to c-Fos regulation, suggesting the existence of important unknown mediators of c-Fos signaling, which need to be identified by further research.

One of the striking findings in this study is the implication of c-Fos/TRAIL pathway in the control of cell necrosis during late infection. To date, *Brucella* are thought of as pathogenic bacteria that do not cause cell death after infection (Gross et al., 2000; Eskra et al., 2003; Chen and He, 2009; Chen et al., 2011). However, recent reports have demonstrated that after successful internalization and after overcoming host defenses, *Brucella* are trafficked to the ER-like compartment where they perform dual functions: replication and self-dissociation. This self-dissociation process was clearly proven to stimulate infected-cell death by apoptosis and necrosis, which are crucial for bacterial egress and dissemination, leading to chronic infection (Turse et al., 2011; Pei et al., 2014). Although this phenomenon was recently confirmed and was shown to be independent of TNF signaling (Hop et al., 2017b), the precise mechanism and pathways that contribute to this process remain unknown. Interestingly, in this study, the c-Fos is the first pathway to be identified that controls cell necrosis but not apoptosis during *Brucella* infection. Furthermore, the suppression of two of the most important downstream molecules, TRAIL and FasL, in c-Fos signaling (Siegmund et al., 2001; Zhang et al., 2007) revealed the involvement of TRAIL in c-Fos-induced necrosis, whereas this process was shown to be independent of FasL. Although the c-Fos/TRAIL pathway is essential for necrotic induction in *B. abortus*-infected macrophages, this contribution is not sufficient (approximately 30% total cell death), strongly suggesting the existence of pathways that remain to be elucidated. Therefore, further investigation of the mechanism via which the c-Fos/TRAIL pathway controls cell death and identification of another pathway could be useful for the design of a therapeutic approach to prevent chronic brucellosis.

In summary, our findings reveal previously unknown roles of c-Fos in the regulation of TLR-4 and IL-10 signaling, which are crucial for *B. abortus* internalization and growth in macrophages, suggesting a similar role for c-Fos in other bacterial infections. Moreover, this study is also the first to determine the role of c-Fos/TRAIL as a host response strategy for bacterial dissemination, causing chronic brucellosis. Finally, investigation of the functions of c-Fos-modulated phagolysomal proteins could provide further evidence and insight into phagosome-bacterial interactions, which are known to be the most important mechanisms for killing intracellular bacterial pathogens.

## Author contributions

HH, LA, AR, and TH carried out all experiments, contributed to data collection and analysis, and participated in drafting the manuscript. LA and SV contributed to revise the manuscript. WM, HL, MR, HC, and SK participated in the design of the study and contributed to revise the manuscript. SK participated in the design of the study, carried out the data analysis, conceived the experiment and prepared the manuscript. All authors read and approved the final manuscript.

### Conflict of interest statement

The authors declare that the research was conducted in the absence of any commercial or financial relationships that could be construed as a potential conflict of interest.

## Supplementary material

Figure S1 c-Fos pathway affects *Brucella* infection. **(A)** RAW 264.7 cells were treated with *c-fos* siRNA and the proliferation activity was examined at different time points. **(B)** Flow cytometry histograms and quantitative analysis of intracellular *B. abortus* growth in *Tnf* or control siRNA-treated cells at indicated time points. **(C)** Cells were treated with or without *Tnf* siRNA prior to infection with *B. abortus*, and the bacterial CFU was determined at the indicated time points. The data represent the mean ± SD of triplicate experiments. The asterisk indicates significant difference (*P* < 0.05).

Figure S2 c-Fos controls B. abortus phagocytosis via TLR-4 signaling. RAW 264.7 cells were treated with *c-fos* siRNA prior to *B. abortus* infection. **(A)** Flow cytometry histograms and quantitative analysis of MAPK activation at 30 min pi. **(B)** Flow cytometry histograms and quantitative analysis of intracellular *B. abortus* growth in cells concomitantly treated with *c-fos* siRNA and anti-TLR-4 mAb. The data represent the mean ± SD of triplicate experiments. The asterisk indicates significant difference (*P* < 0.05).

Figure S3 c-Fos/TRAIL pathway regulates cell necrosis during *Brucella* infection. RAW 264.7 macrophages were treated with different siRNAs prior to *B. abortus* infection and subjected to staining with apopxin green (apoptosis) or 7-AAD (necrosis). **(A)** Flow cytometry histograms of apoptosis and necrosis from the indicated cells at 48 h pi. **(B)** Flow cytometry histograms of cell apoptosis and necrosis from the indicated cells at 48 h pi.
