# Supplementary figures and images for: The Key Role of c-Fos for Immune Regulation and Bacterial Dissemination in Brucella Infected Macrophage
Source: Front Cell Infect Microbiol. 2018 Aug 21;8:287. doi: 10.3389/fcimb.2018.00287 (PMC6110913; doi:10.3389/fcimb.2018.00287)

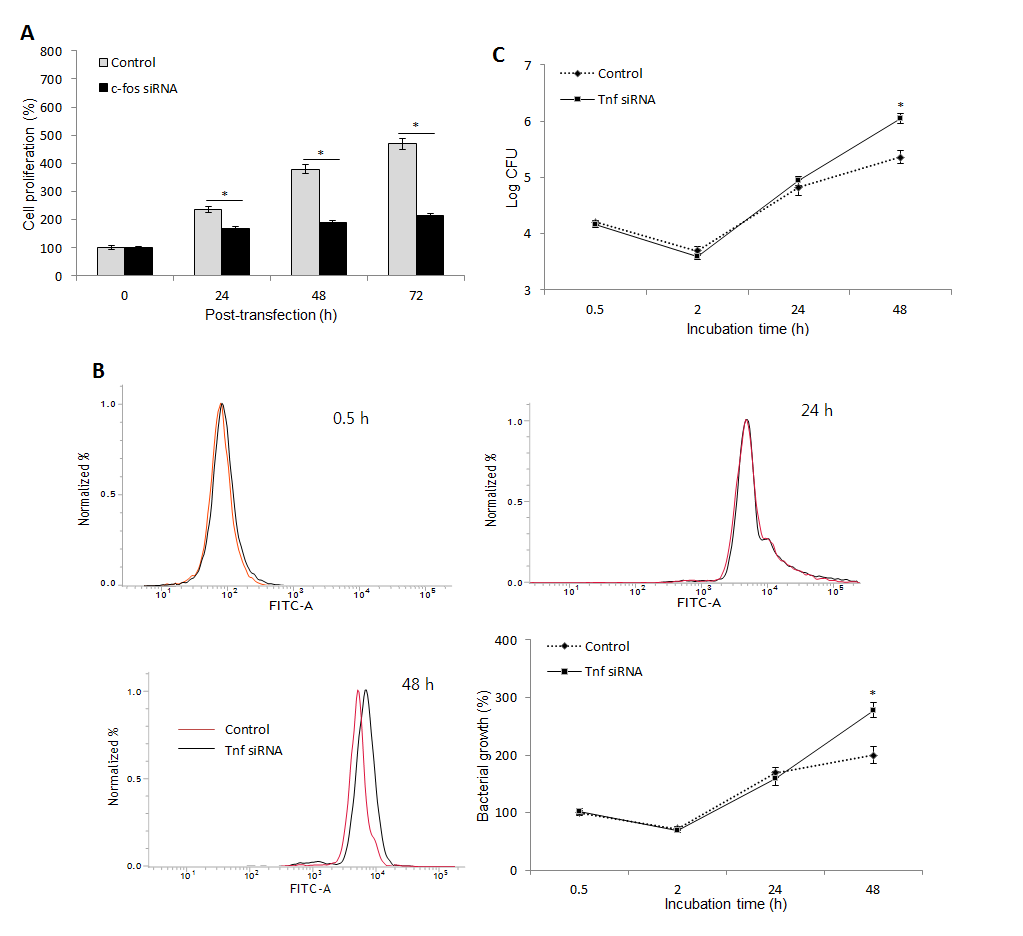

Supplement: Figure S1 — c-Fos pathway affects Brucella infection. (A) RAW 264.7 cells were treated with c-fos siRNA and the proliferation activity was examined at different time points. (B) Flow cytometry histograms and quantitative analysis of intracellular B. abortus growth in Tnf or control siRNA-treated cells at indicated time points. (C) Cells were treated with or without Tnf siRNA prior to infection with B. abortus, and the bacterial CFU was determined at the indicated time points. The data represent the mean ± SD of triplicate experiments. The asterisk indicates significant difference (P < 0.05). [file Image_1.TIF]

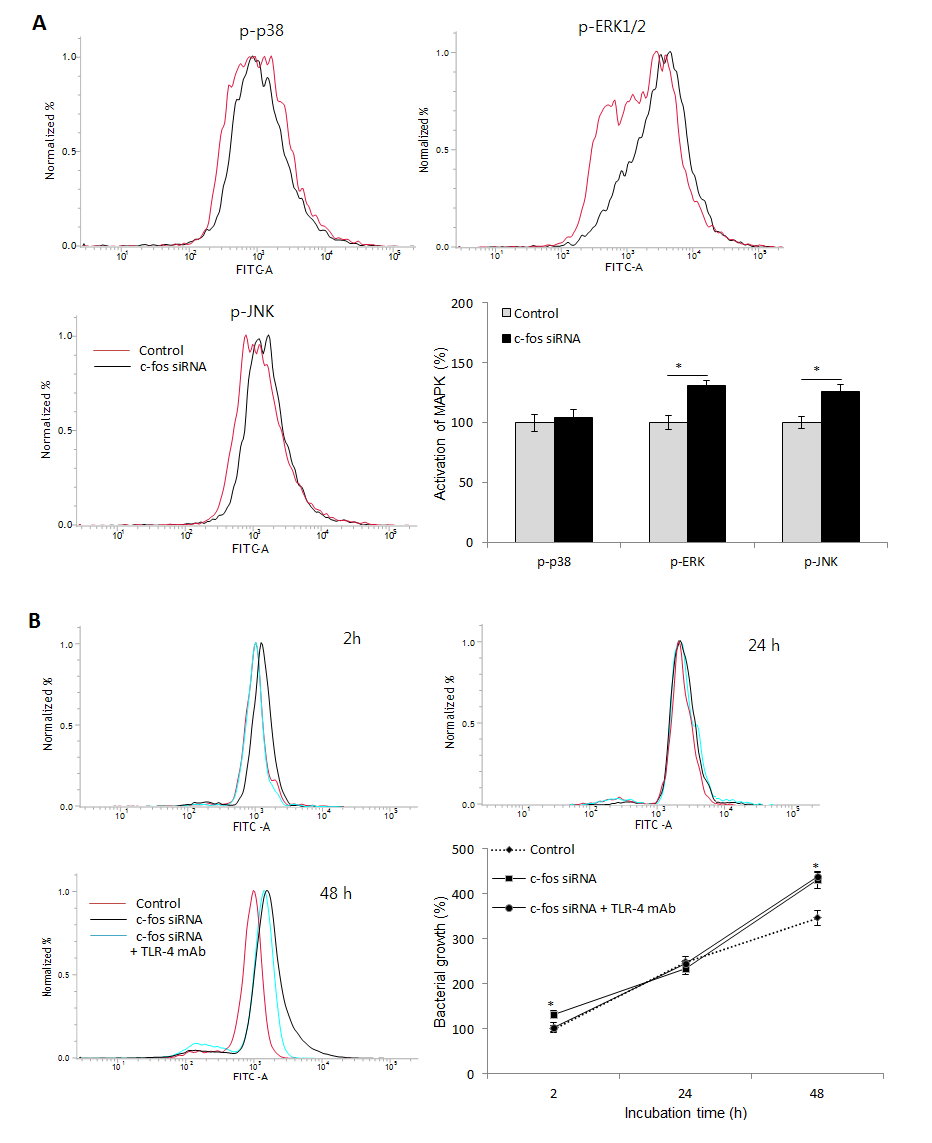

Supplement: Figure S2 — c-Fos controls B. abortus phagocytosis via TLR-4 signaling. RAW 264.7 cells were treated with c-fos siRNA prior to B. abortus infection. (A) Flow cytometry histograms and quantitative analysis of MAPK activation at 30 min pi. (B) Flow cytometry histograms and quantitative analysis of intracellular B. abortus growth in cells concomitantly treated with c-fos siRNA and anti-TLR-4 mAb. The data represent the mean ± SD of triplicate experiments. The asterisk indicates significant difference (P < 0.05). [file Image_2.TIF]

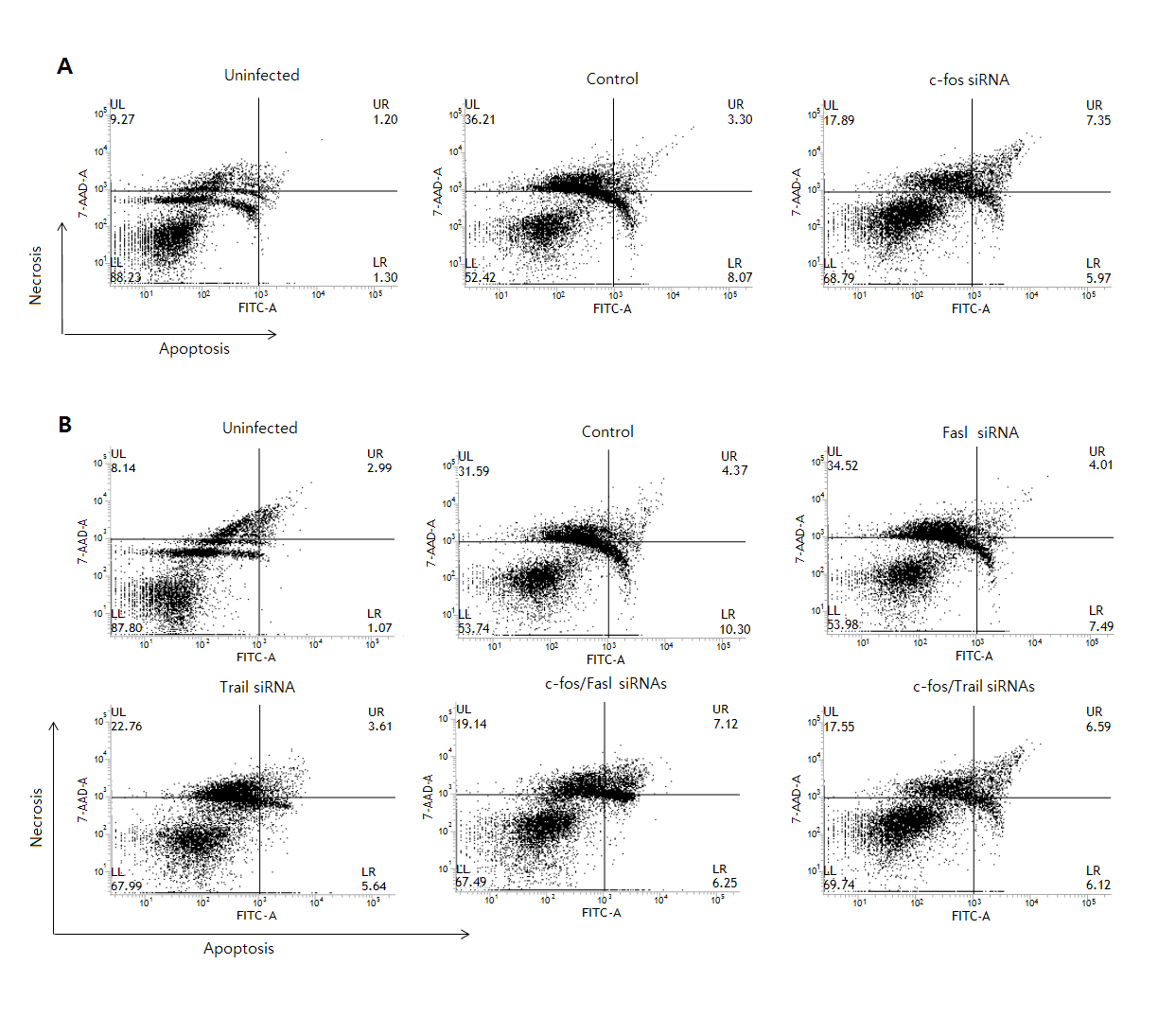

Supplement: Figure S3 — c-Fos/TRAIL pathway regulates cell necrosis during Brucella infection. RAW 264.7 macrophages were treated with different siRNAs prior to B. abortus infection and subjected to staining with apopxin green (apoptosis) or 7-AAD (necrosis). (A) Flow cytometry histograms of apoptosis and necrosis from the indicated cells at 48 h pi. (B) Flow cytometry histograms of cell apoptosis and necrosis from the indicated cells at 48 h pi. [file Image_3.TIF]
